# Cultural and ethnobotanical legacy of native potatoes in Colombia

**DOI:** 10.1186/s13002-022-00557-1

**Published:** 2022-09-19

**Authors:** Daicy Yaneth Galvis-Tarazona, Zaida Zarely Ojeda-Pérez, Diana Marcela Arias-Moreno

**Affiliations:** Grupo de Investigación BIOPLASMA, Facultad de Ciencias, Universidad Pedagógica y Tecnológica De Colombia (UPTC), Avenida Central del Norte, 050030 Tunja, Boyacá Colombia

**Keywords:** Native potatoes, Conservation, Cultural importance, Ethnobotany, Traditional knowledge

## Abstract

**Background:**

Native potatoes are Andean tubers of great historical, social, food, genetic and nutritional importance, and they contribute significantly to food security by supplementing the household diet and also providing alternative income. Even when their cultivation and consumption imply great benefits, their use and local preservation depend to a large extent on the recognition of their ethnobotanical and cultural importance. In this context, this study consolidates an important ethnobotanical research bases for native potatoes in Colombia.

**Methods:**

The study collected data through semi-structured interviews and dialogues (130) in the municipality of Chiscas, department of Boyacá, central-eastern Colombia. The questionnaire was focused on native potatoes and sought to investigate the knowledge related to cultivation, diversity, patterns and forms of preparation for use and consumption. Likewise, knowledge heritability mechanisms were investigated and ethnobotanical indices of relative importance, use and culture were estimated.

**Results:**

Documentation of ethnobotanical knowledge included aspects such as seed care and availability, cultural management of the crop, patterns of use and consumption, as well as ways of preparing the tubers. In total, 23 vernacular names of native potato and 360 reports of use (commercial, domestic or ritual-magical) were recorded for the 15 main genotypes. Quantitative estimates included the importance index: (a) cultural, for which values ranged between 0.059 and 0.812; (b) relative, with records between 0.04 and 0.43; and (c) use, which ranged between 0.06 and 0.63. The ethnobotanical importance index (d) for native potatoes was 57.26, which corresponds to a “very high” ethnobotanical value. This allowed us to identify that *Criollas* were the most recognized and used potatoes within the community. In addition, it was shown that vertical transmission is the main way in which traditional knowledge about native potatoes is inherited. Finally, an artificial intelligence tool was preliminarily implemented to identify the polarity generated in the interviewees by the questions.

**Conclusion:**

The results of this research provide valuable information on the ethnobotany of native potatoes in Colombia. The genotypes used by the community of the municipality of Chiscas were recognized for their high gastronomic and nutritional potential, as well as for their great ethnobotanical and cultural importance. These data can be considered as a valuable tool to support any action aimed at the conservation and revaluation of these tubers.

## Background

Potato (*Solanum tuberosum L.*) is considered as a crucial agricultural resource for mankind during the last eight centuries, for its contributions in terms of food security, nutrition, population growth and urbanization in many regions [1]. Potato is the most important non-cereal crop in the world and an invaluable staple food with high biological and genetic diversity that includes wild, native and improved varieties [2]. The history of these tubers indicates that their cultivation dates back more than 7000 years and that their center of origin is located near Lake Titicaca, a border area between Bolivia and Peru [3].

Ancestral Andean farmers domesticated the potato through selection processes based on wild and native materials; genetic resources that present different shapes, colors and sizes [4]. Native potatoes are a legacy of the Andean civilization to the heritage of agricultural, food and industrial biodiversity and are a potential source of genes for the improvement of commercial varieties [5–8].

According to the International Potato Center (CIP), there are more than 4000 native genotypes or landraces, which have been conserved mainly by producers, in in situ germplasm banks [9]. In the Andes, native potatoes are grown from western Venezuela to central Bolivia, at altitudes ranging from 2000 to 4200 m. Traditionally, they have been a source of food, of great nutritional value, which guarantees food security, in Andean communities, and have therefore formed an important part of their cultural identity [10].

Research related to native potatoes has been mainly oriented to the characterization of the morphological, the genetic and physicochemical variation of the genotypes, the evaluation of the agroindustrial potential and biological functionality of their components, as well as their possible use in genetic improvement programs [11–16], on the other hand, although the interaction of human communities with native potatoes has been studied worldwide [17–20].

In Colombia Boyacá is one of the departments with the highest potato production and consumption. For these, some studies have been reported that address agronomic, anthropological, ethnobotanical (descriptive), morphological and generic uses of native potatoes [21–24]. To the best, no detailed simultaneous quantitative and qualitative determination of the ethnobotanical and cultural value of native potato genotypes in this country has been reported. In addition, for Boyacá there are no known reports on genetic diversity, identification of traits of agronomic interest or for crop breeding.

Ethnobotany is a discipline that allows to understand patterns of plant use in human groups, and traditional knowledge associated with the usefulness of plants is highly dependent on the local context [25]. Therefore, it is essential to conduct research in different socio-cultural groups, since the development of this type of studies provides useful information for the reevaluation, rescue, care and conservation of ancestral resources [26].

Consequently, the objective of this study was to document the traditional and ethnobotanical knowledge of native potato genotypes grown in the municipality of Chiscas (Boyacá, Colombia). Thus, the following hypothesis was raised: The inhabitants of the municipality of Chiscas use the native potato genotypes, with greater recognition for cultivation and family consumption. This legacy has been preserved through the transmission of knowledge from generation to generation and is represented by cultural and ethnobotanical values.

## Methods

### Study area

**Fig. 1:**
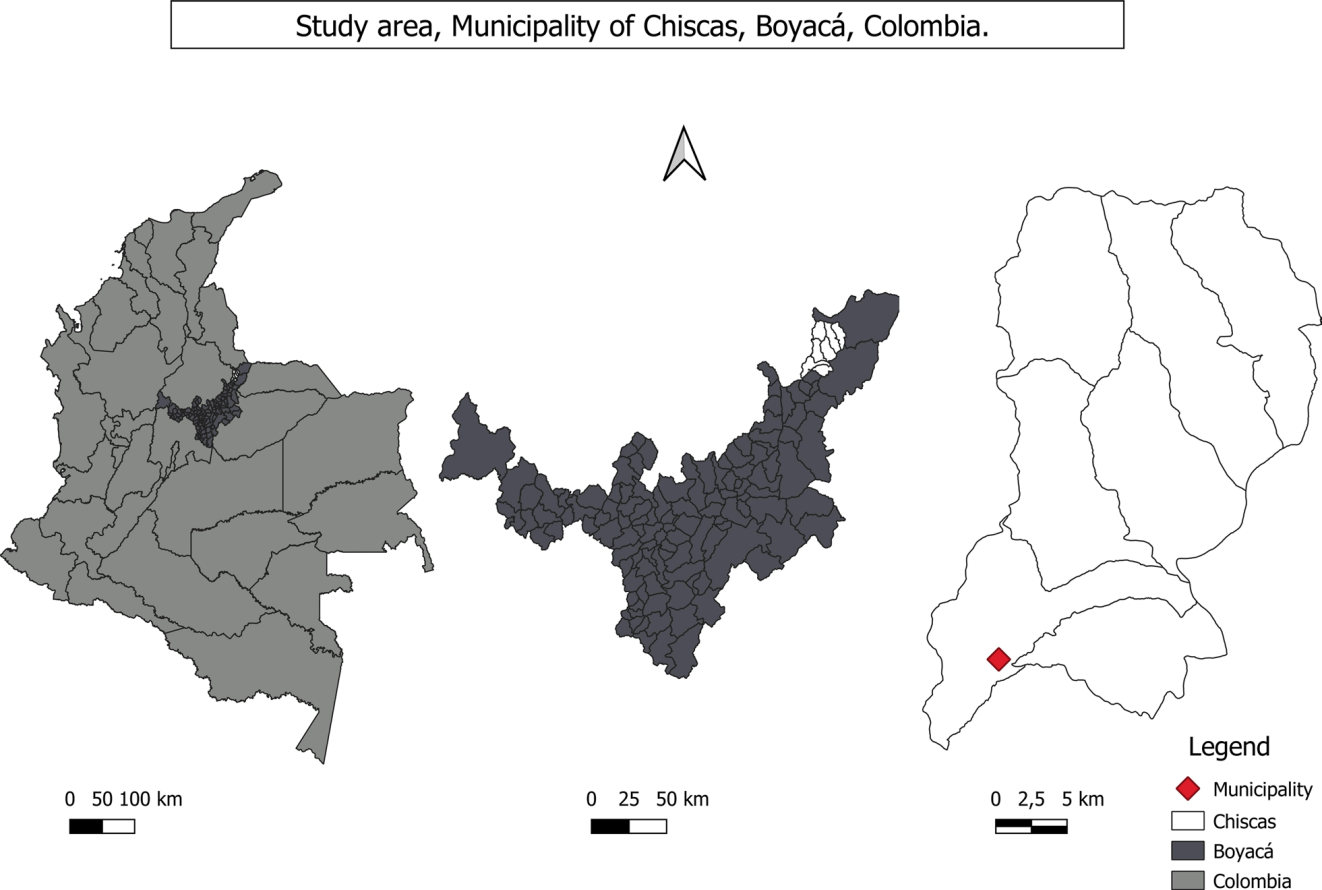
Study area, Municipality of Chiscas, Boyacá, Colombia

One of the communities that still protect and cultivate native potatoes is located in the municipality of Chiscas, in the department of Boyacá, Colombia. The town has an area of 662,732 km^2^ and is located in the eastern mountain range to the northeast of the department of Boyacá at 6°33′11″ N, 72°29′58″ W (Fig. 1). Its name is derived from the ancient settlers known as the Laches and Chiscas who described this territory as a muddy place [27]. The municipality is divided into nine sectors and has an estimated population of 3.587 inhabitants [28]. Average annual rainfall ranges between 1200 and 1500 mm, with rainy periods interspersed with a dry period and another slightly rainy one. Most of the villagers, including the youngsters, speak Spanish [27]. The 2018 multidimensional poverty index indicated that about 60% of the municipality’s population was at some level of poverty [28].

### Field survey and data collection

To develop the present study, it was necessary to design the questionnaire and collect information. The data obtained in the interviews served to carry out a sentiment analysis, to quantitatively determine the transmission of knowledge and the importance of native potatoes. In this last aspect, it is important to point out that widely known ethnobotanical indices were estimated, such as relative importance, use value, ethnobotanical importance and cultural importance. Additionally, the relationship by type of use was made for each of the reported genotypes.

The native potato genotypes referenced here belong to the Andigena group, taxonomically classified as *Solanum tuberosum* ssp. *andigena* group. Their detailed botanical description can be found in the book entitled: “Colors and flavors of my land: native potatoes grown in Boyacá” [21].

### Questionnaire design

This study was based on semi-structured interviews (exploratory interview that follows a previously devised guide or protocol and focuses on a central topic to provide a general structure and also allows for additional topics to be addressed as the conversation unfolds) [29]. The questionnaire used was empirically validated through a pilot test with 55 inhabitants of the El Sote sector in the municipality of Motavita, Boyacá. In this area, potato cultivation is historical and recognized. The first version of the questionnaire was given to the participants to assess the comprehension, reliability and reproducibility of the questions. Considering the practical work in the field, the adjustments required, fundamentally related to a non-understanding of some terms, were made.

### Collecting information

The community of the municipality under study is made up of a rural area and an urban nucleus. Since the study sought to understand the historical dynamics of use and transmission of knowledge related to native potatoes, tours, visits and interviews were carried out in both sectors.

Fieldwork included semi-structured interviews, key informant conversations, participant observation, and dialogues (process of interaction which can be applied to different conditions and circumstances) [30]. In total, 130 informants were selected using the snowball method. At first, before any interview, we introduced ourselves to the people in charge of the town; after their agreement, the participants were asked if they could be interviewed in the context of our study. They were free to participate or not; that is, their inclusion was voluntary.

During the field research, each informant listed the native potatoes he/she remembered, including current and past information. The interviews consisted of two parts, the first part recorded data on the basic situation of the informants (age, education, occupation), and the other part included questions related to the cultivation, consumption and commercialization of native potatoes including among others, local name, availability of seeds, type of use, part used, frequency of consumption, gastronomic properties and other uses. The designed form was applied with the help of the tool for cell phones and computers, KoBoCollect v.1.25.1 [31].

### Quantitative ethnobotany

The ethnobotanical importance of native potato species in the studied community was assessed by quantifying the historical ethnobotanical indices listed below.

### Uses and importance of native potato genotypes

To understand and contrast the relationship between types of uses for each native potato genotype mentioned by the participants, the ethnobotanyR package proposed by Whitney [32] for the R software was used. The string diagram was used to create the corresponding graph, and the cultural value index was estimated for the 15 most mentioned genotypes (*ethnobotanyR*). The determination of the index is based on the formula and the factors proposed by Reyes-García [33].

### Relative importance

This index reflects the relative frequency of citation and the relative number of categories of use. The value obtained allows to present the most versatile native potato genotypes. The relative importance index varies theoretically from 0, when no use of the plant is mentioned, to 1, when it is mentioned most frequently as useful in the maximum number of use categories. It was estimated using the formula proposed by Tardío and Pardo-De Santayana [34].

### Use value

This index takes into account the uses mentioned by the participants for each of the native potatoes. For this index, values between 0 and 1 can be obtained, with 1 being the highest value, associated with high utility. This was estimated using the formula proposed by Prance et al. [35].

### Ethnobotany importance

The ethnobotanical importance value index of native potatoes was estimated based on the formula proposed by Lajones-Bone and Lema-Tapia [36], with some modifications. In other words, although the minimum (0) and maximum (100) values were maintained, the cultural elements, each of their categories and weight within the formula, were adjusted taking into account uses and agroeconomic characteristics within the local context under study.

Therefore, for the estimation of this index, four quantitative qualifiers were used, as follows. (1) CALPARE: associated with the part of the plant used, that is, stems, leaves, flowers or tubers. (2) CALSURE: refers to the uses those potatoes are given, including commercial, domestic, medicinal, ritual or magical, or others. (3) CALEXC: related to the size of the crop expressed in hectares and that could be > 10, 8–10, 6–7.9, 4–5.9, 2–3.9, 0.1–1.9. (4) CALTIC: referring to the time, in years, that the potato genotypes have been cultivated, the values could be included within the following ranges: 9–10, 7–8, 5–6, 3–4, 1–2.

### Cultural transmission

The dynamics, most frequently used for the transmission of this knowledge, was evaluated for each age group (Table 1). For this, the percentage of cultural transmission will be reduced based on the answers issued by the participants regarding who taught them what they know about these tubers. To do this, the percentage of cultural transmission was deduced based on the answers given by the participants regarding who taught them what they knew about these tubers. Five optional responses were given: grandparents, parents, siblings, uncles or acquaintances, which were the most cited during the validation of the questionnaire. For the estimation of the percentages and the graphical representation of the results, R [37], was used.

**Table 1:** Age range of the interviewees

General data	Number of participants
*Age range (years)*	
10–19	24
20–34	24
35–59	26
> 60	29

### Sentiment analysis

In artificial intelligence, the use of methodological tools such as the training of specialized data mining algorithms allows identifying the sentiments expressed by people in different scenarios (polarity). For this reason, in this study, a sentiment analysis was carried out, which allows the perceptions of the participants to be categorized as positive, negative or neutral. The analysis was carried out by the company Quaxam Datalab, based on the text information obtained from the interviews.

For this purpose, and based on natural language processing (NLP), the software products QUnderstand and QFeel were used for the extraction of sentiments and emotions. The process comprised three stages: In the first stage, the text was sequentially adapted, in stage 2, the information was processed and the unstructured data were extracted, and in stage 3, a model was built to summarize the results obtained.

The explorations were carried out from two approaches, the first (a) identified and individualized the sentiments generated in the interviewees when approaching different topics and the second (b), the emotions generated in the interviewees were recognized and categorized with respect to the totality of the questions raised during the interview. The results of the analysis take values represented on a polarity scale ranging from − 1 (totally negative sentiment) to 1 (totally positive sentiment). Thus, values between 0.2 and − 0.2 on the scale allow us to infer a neutral sentiment.

## Results

### Ethnobotany information

For the inhabitants of the municipality of Chiscas, native potatoes are a fundamental food, and a historical and cultural legacy, which they hope will be revalued in today’s society. Farmers are their main conservationists and their availability and preservation depend on them, both to start a new cultivation process and for marketing and local and regional consumption. The seeds (tubers) are protected mainly in the rural area of the municipality, where they are mostly grown.

After harvest, the tubers are stored until they develop sprouts. Then, from this, they are sown in prepared land (depending on the extension by means of a plow or tractor; in this activity the furrows are opened in the land and the soil is removed). The care and cultural management of the crop commonly includes weeding (the removal of weeds and other plants other than those planted) and hilling (in which the loose soil is brought closer to the plant). Additionally, these activities can be complemented with organic fertilization.

**Fig. 2:**
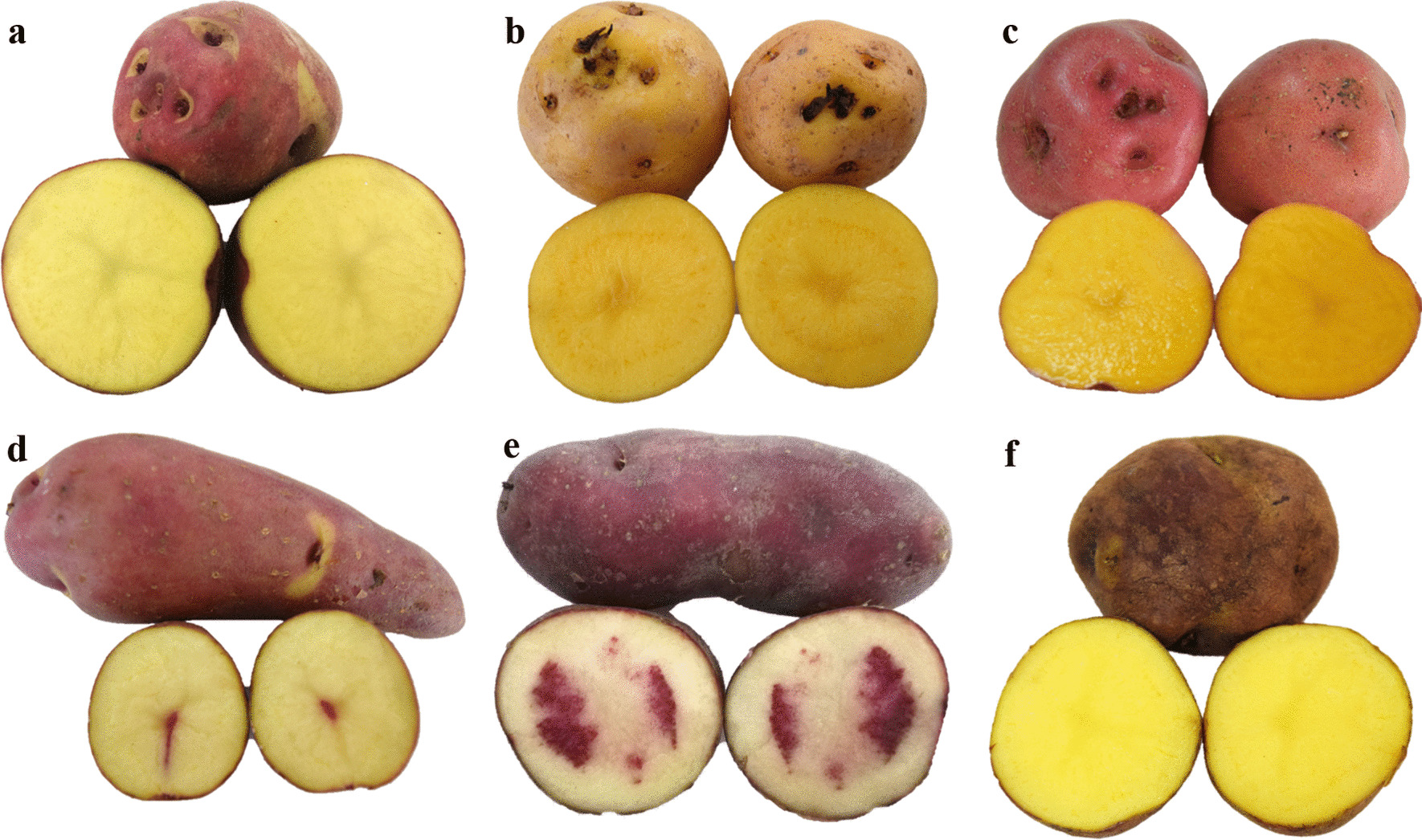
Representative images of native potato tubers grown and used by the community of the municipality of Chiscas, Boyacá, Colombia. **a** *Banderitas*; **b** *Criollas*; **c** *Manzana*; **d** *Palo de Árbol*; **e** *Pepinas* and **f** *Rosadas*

In the community, native potatoes were described as different and unique in characteristics such as flavor, shape, size and texture. Also, they are known with striking names which in some cases come from the morphological characteristics of the tubers or plants and in others from their place of origin (Fig. 2). Thus, *Argentinas*, *Arrayanas*, *Banderitas*, *Barrosa*, *Bola de Toro*, *Colinas*, *Cóngolas*, *Criollas*, *Gramapollo* (*Pico de pollo*), *Manzana*, *Mariposa*, *Marucha*, *Monserrata*, *Moradas*, *Negra*, *Palo de Árbol*, *Pana Azul* (*Azules*), *Pana Blanca*, *Paño de Manos* (*Marranito*), *Pepinas* (*Pepina Rodeo*), *Rojas*, *Rosadas* and *Tocana*, integrated the list of cited vernacular names.

**Fig. 3:**
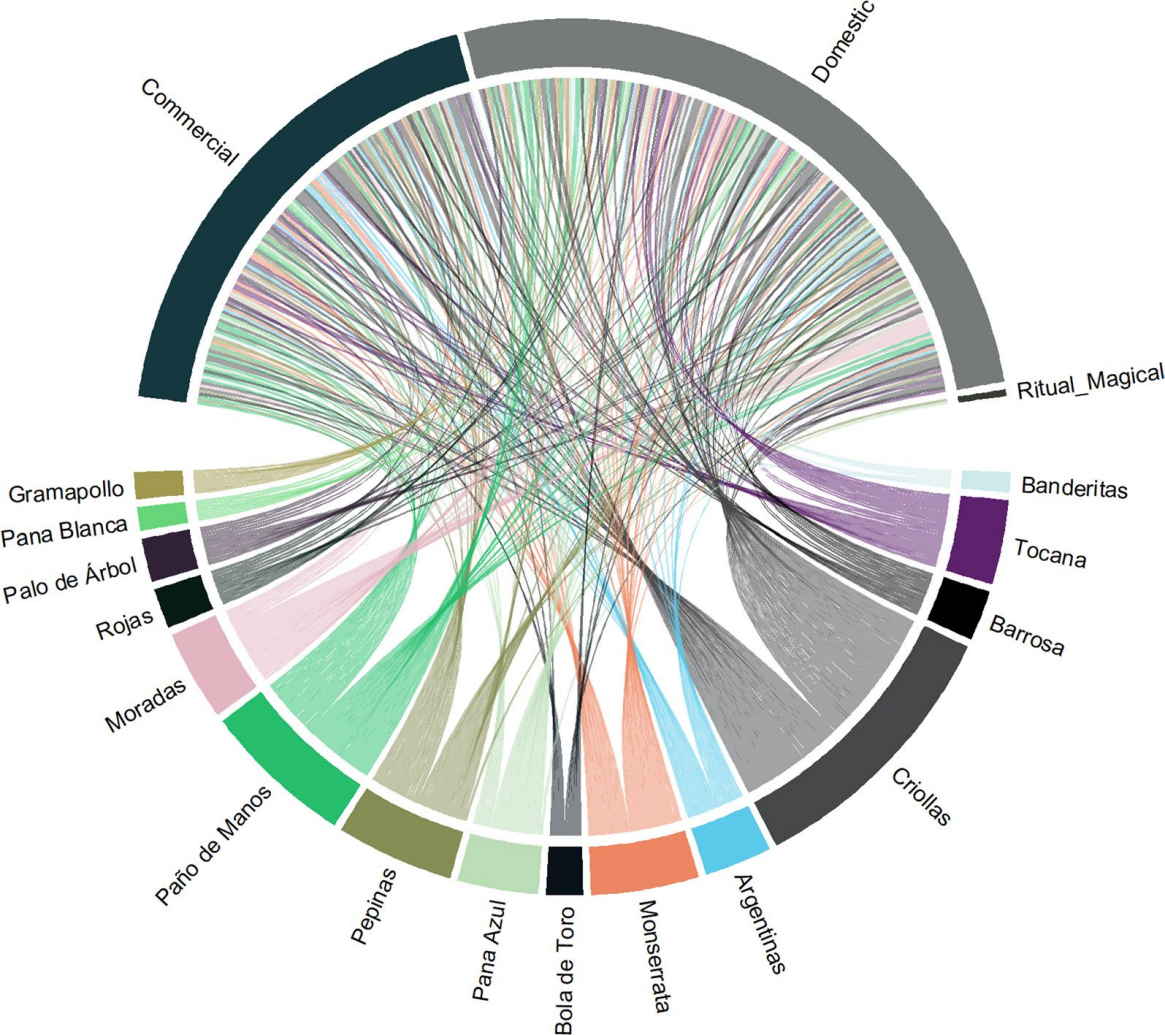
Chord diagram of distribution of 360 use reports among 15 genotypes of native potato and three categories of use in Chiscas, Boyacá, Colombia. The use categories are shown in top half of circle and the native potato genotypes are located from the bottom half

Native potato tubers have been used in Chiscas for different purposes. Thus, for the 15 most cited genotypes, 360 reports of use were referenced for the commercial, domestic, and ritual or magical categories (Fig. 3). The use in homes was the most recognized typology and each genotype has a specific pattern of use. For example, Corduroy Blue, Purple and Red potatoes are mainly destined for domestic use, while *Criollas* and *Argentinas* are destined in parallel for commercialization and domestic use (Fig. 3).

The consumption of native potatoes was directly related to the nature of the crop. Thus, according to what was mentioned by the interviewees, the usual frequency of intake immediately after the harvest is daily and then decreases as resources are depleted. Another factor recognized as conditioning was the duration of production cycle, by implying a period greater than that required for the development and maturation of the tubers, since this determines their availability for use.

The ways of preparation of native potatoes are considered diverse and the aim is to accentuate the organoleptic properties, allow the preservation of the nutraceutical value, and/or give added value to the recipes (color and flavor). Thus, with respect to “cooked potatoes,” it was recognized that they can be prepared: saladas (with skin), rayadas (half of the tuber skin is removed) and peladas. (The entire skin of the tuber is removed.) They have also been used to obtain three types of “potatoes fries”: chips, French (sticks), and hulls (with skin). In addition, they have been important components of caldos, soups, sancochos and other dishes. They can also be baked and used to make pasteles, empanadas, salads (ground), purés (macerated) and Spanish cake.

### Quantitative ethnobotany

#### Cultural importance

**Fig. 4:**
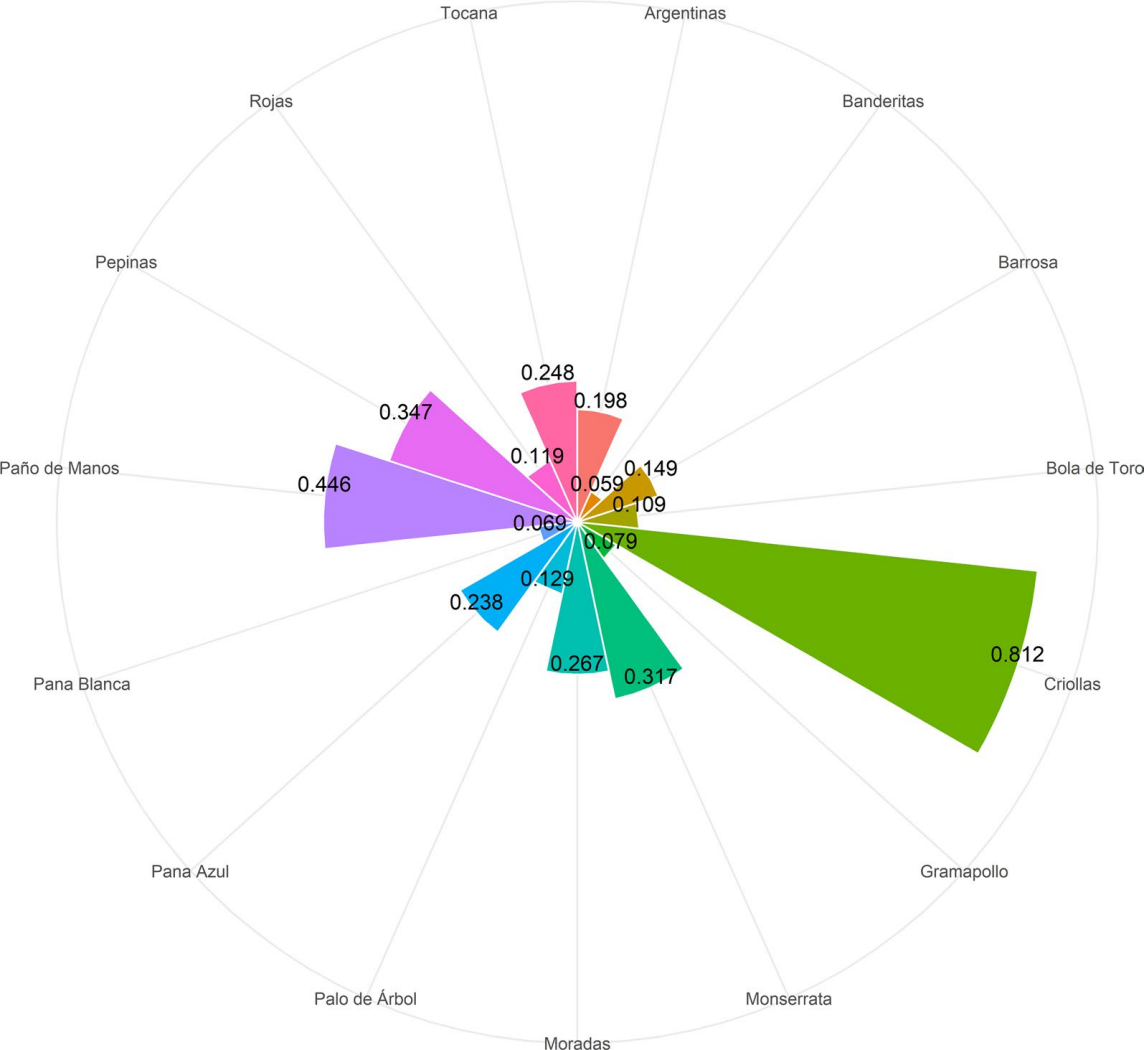
Radial bar chart of cultural importance index of 15 native potato genotypes of the municipality of Chiscas, Boyacá. The index value of each genotype is displayed as a circle segment with radius proportional to the value

The cultural value of native potatoes was evidenced not only by their varied uses in meeting human needs such as health and food, but also by the quantitative values determined for the index, which ranged between 0.059 and 0.812 (Fig. 4). For *Criollas, Paño de Manos, and Pepinas*, the highest estimates were obtained (0.812, 0.446 and 0.347, respectively) (Fig. 4). Its significance and with it the difference identified between the genotypes can be explained by the recognition within the community and the number of uses that were associated with each of them.

### Ethnobotanical importance

In addition to the cultural value, in this study the indices of: relative importance, use value and ethnobotanical value were also determined. The results determined for the first two are summarized in Table 2. In this, the 15 main genotypes were ordered by merit. The relative importance ranged between 0.04 and 0.43, the use value from 0.06 to 0.63. The potatoes with the highest index values were *Criollas, Paño de Manos, Pepinas, Moradas, and Monserrata*. Unlike *Pana Blanca* and *Banderitas* that had the lowest index values, being less mentioned.

**Table 2:** Relative importance value and uses of the fifteen genotypes most commonly mentioned by the informants

Vernacular name	No. of citations	Relative importance	Use value
*Criollas*	56	0.61	0.63
*Paño de manos*	29	0.32	0.35
*Pepinas*	22	0.24	0.27
*Moradas*	22	0.23	0.21
*Monserrata*	19	0.21	0.25
*Pana Azul*	17	0.18	0.17
*Tocana*	16	0.18	0.19
*Argentinas*	13	0.15	0.16
*Barrosa*	10	0.11	0.12
*Rojas*	9	0.10	0.09
*Palo de Árbol*	8	0.09	0.10
*Gramapollo*	7	0.07	0.06
*Bola de Toro*	6	0.07	0.08
*Pana Blanca*	4	0.05	0.07
*Banderitas*	4	0.04	0.06

On the other hand, in the quantification of the index of ethnobotanical importance, for the estimators, minimum values of 14.86 and maximum values of 80 were obtained; therefore, the final value obtained for the index was 57.26. This, according to the interpretation given by Lajones-Bone and Lema-Tapia [36], would suggest that native potatoes represent a “very high” ethnobotanical value in the municipality of Chiscas, Boyacá.

### Cultural transmission

**Fig. 5:**
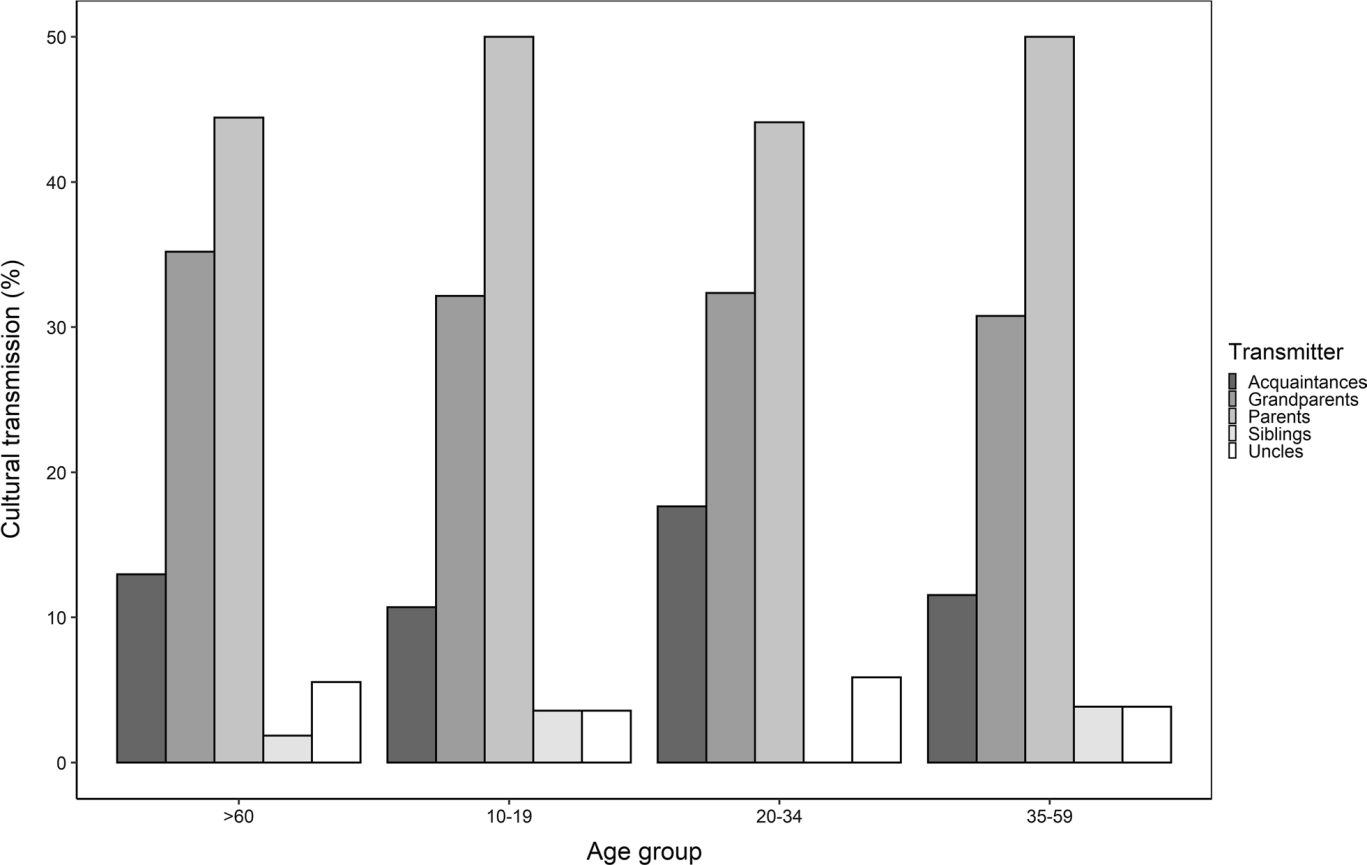
Transmission of cultural knowledge by age groups associated with the use and cultivation in the community of Chiscas, Boyacá

The cultural tradition and the knowledge derived from the interaction of the community of the municipality of Chiscas with the native potatoes, have been preserved and enriched for several generations. This fact was recognized in the dynamics of transmission of knowledge, since about 80% of the older adults (> 60 years old) interviewed declared that their knowledge was inherited from their parents and grandparents. The same reply was also given by more than 30% of the interviewees categorized in the three remaining age groups (Fig. 5). Thus, vertical transmission has resulted in a cultural legacy, complemented to a lesser extent by the transfer of knowledge between individuals of the same generation (horizontal transmission). Although a large part of the interviewees indicated that their predecessors cultivated these tubers, this proportion decreased significantly when asked about the development of this activity today.

The local connoisseurs of the crop expressed the importance of the inherited knowledge regarding the selection of the land and the “seeds.” Planting in rainy seasons and cultural management activities such as weeding and hilling have also been traditionally preserved.

### Sentiment analysis

**Fig. 6:**
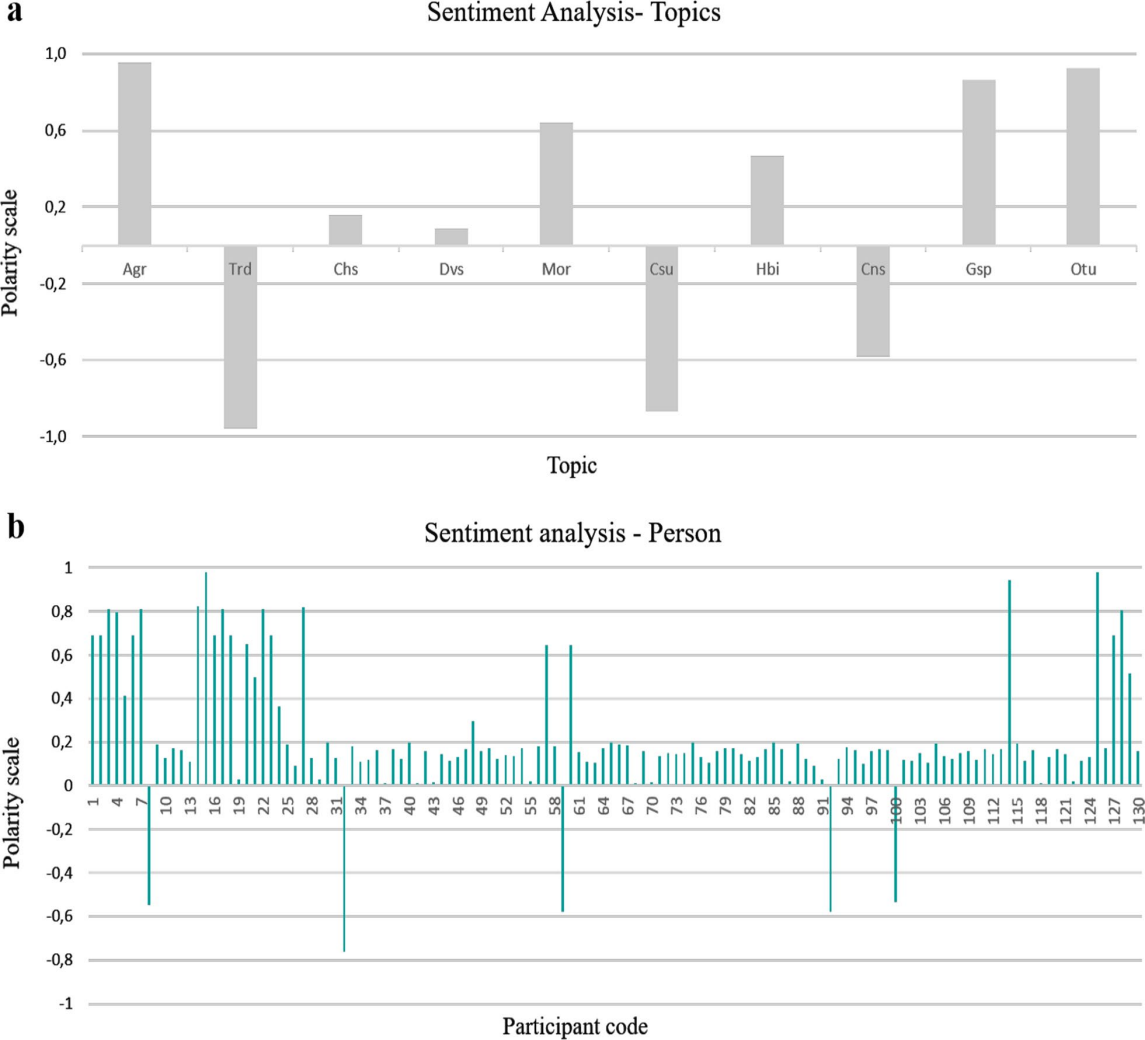
Sentiment analysis per topic (**a**) and person or interviewers (**b**). *Agr* agronomy, *Trd* trade, *Chs* crop history, *Dvs* diversity, *Mor* morphology, *Csu* consumption, *Hbi* heritability *Cns* conservation, *Gsp* gastronomic potential, *Otu* other uses

In the first approach to sentiment analysis developed, ten main themes discussed in the interviews were identified (Fig. 6a). These included: agronomy, trade, crop history, diversity, morphology, consumption, heritability, conservation status, gastronomic potential and other uses. Positivity was associated not only with the agronomic, gastronomic and use potential that native potatoes represent for the participants, but also with knowledge about the morphological characteristics of these tubers and the importance of inheriting knowledge related to these resources.

On the other hand, the items that generated a negative sentiment are associated with activities that are not carried out effectively, such as trade and conservation. This perception can also be explained by the fact that 62% of the interviewees indicated that they noticed a reduction in the morphotypes of native potatoes in the locality.

Additionally, the polarity obtained compared to the general questionnaire was positive and neutral in greater proportion, with a percentage value close to 96 (Fig. 6b), since only in five of the interviewees did the questions addressed generate a negative perception. It is necessary to mention that its connotation did not become highly unfavorable. This result may be associated with partial ignorance or low interest in the topic addressed: “native potatoes.”

## Discussion

### Ethnobotany knowledge and importance associated with the cultivation and consumption of native potatoes

Ethnobotany studies the way in which human communities have been able to satisfy their material and spiritual needs through their relationship with plants [38]. From these relationships, knowledge and customs have been generated that are essential for the development of social groups [39, 40]. For example, in this study it was found that native potato genotypes have been used for different purposes.

The utilization for domestic purposes was associated here in all genotypes. However, potatoes referenced as *Criollas, Paño de Manos* and *Pepinas* have also been marketed locally. These use patterns represent the agronomic cosmovision of Andean communities and can be derived from the exchange of knowledge between different members of the community [41].

Furthermore, the information obtained through semi-structured interviews and dialogues, i.e., with direct participation of the community, is considered of great importance for the preservation of agrobiodiversity [42], as well as necessary for the planning and development of effective strategies aimed at the sustainable use of resources [43]. It also served as a basis for the implementation of quantitative ethnobotany tools.

Noteworthy, the estimation of the different variables made it possible to consolidate precise, relevant, and consistent inferences throughout all phases of the study. The indices used made it possible to accurately determine the importance of native potatoes for the community of the municipality of Chiscas, Boyacá, Colombia. In this context, the data obtained for the cultural importance index for native potato genotypes were superior to those reported by Whitney et al. [44], for ethnospecies grown in gardens in Uganda. *Criollas*, *Paño de Manos* and *Pepinas* were recognized as genotypes of greater traditional relevance for the Chiscan community.

Moreover, the relative importance values determined for the native potato genotypes, which varied from 0.02 to 0.95, are comparable to those reported in research conducted by Bussa & Belayneh [45], Faruque, et al. [46], Lara Reimers et al. [47] and Ranfa and Bodesmo [48]. Therefore, it can be affirmed that the genotypes for which the highest values were related are prevalent within the community and have a high transversality in the use categories evaluated. With this, it was we determined that *Criollas* potatoes are the most recognized and versatile group within the community.

The use values determined here are related to those reported by Gonçalves dos Santos et al. [49] for crops such as corn, lemon, orange, mango and beans. Also, they were lower than the estimates by Paiva de Lucena et al. for native flora [50] and for species with high medicinal and nutritional potential [51], may be due to the differential utility of species or genotypes. In other words, while in some cases only one part of the plant is used, in others, different parts allow different needs to be met in the community.

Additionally, for the community of the municipality of Chiscas, native potatoes represent a very high ethnobotanical value [36]. Therefore, the applied methodology made it possible to determine the cultural and ethnobotanical value of these tubers. It should also be noted that, here, the strengthening of the dynamics of oral tradition could contribute to the preservation of this great cultural legacy.

*Criollas* potatoes were the genotype that obtained the most significant values for all the quantified indices, as it was the genotype most preferred by Andean communities since it is legendary and has been associated with their agronomic and nutritional attributes [52]. Also, their organoleptic properties and agroindustrial potential have promoted the acceptance of these potatoes by companies and consumers [53].

### Perception of the agronomic, gastronomic and use potential of native potatoes

The sentiment analysis conducted in this study consolidates important practical bases that can be considered within ethnobotanical research, as its application enabled by the “transcribed statements” confirmed the perceptions identified during the field work.

In a first approach, the feelings generated when assessing some key aspects related to native potatoes were determined. For example, the perception of the development of cultural events (local or regional) in which the use and consumption of native potatoes was promoted was categorized under a negative perception, related to the limited realization of such events.

However, in the second approach, neutrality was predominant, may be due to the fact that most of the questions addressed were conceptual or required prior knowledge. Such questions, i.e., those based on “which, how many or where,” have a high potential to generate a neutral sensation or feeling [54].

In summary, it is expected that the scientific background obtained in this study can guide new multidisciplinary research scenarios focused on native potatoes. It is also expected to arouse, in governmental sectors, the interest in conserving these valuable genotypes rarely available in national trade and of great importance for food security in the rural sector.

In addition to this, the development of this type of studies aims to promote consumption, cultural reevaluation, and the exploitation of the gastronomic and nutraceutical potential of native potatoes. Likewise, it is suggested that agroindustrial exploration could allow obtaining value-added products. This could lead to the establishment of native potato production chains to improve the quality of life of the country’s producers.

## Conclusion

This research allowed the consolidation of a detailed ethnobotanical report for the native potatoes of Colombia. In this report, the ethnobotanical and cultural importance is detailed through two perspectives. Firstly, the traditional knowledge related to cultivation, consumption and forms of preparation was characterized, whereby the great ancestral legacy that these genotypes represent for the communities of the country was evidenced. Secondly, the most recognized and important genotypes were identified and the most relevant knowledge transmission dynamics were determined. Together, the results obtained made it possible to recognize aspects that should be strengthened to guarantee the biological and cultural preservation of native potatoes, through the promotion of their cultivation, consumption, and scientific and agroindustrial use. Finally, it is hoped that this study will constitute a crucial reference for the development of similar analyses oriented to other genetic resources and/or ethnic communities.

## Data Availability

All data generated or analyzed during this study were included in this published article (along with supplementary files). And the online version containing supplementary material will be available at: https://drive.google.com/drive/folders/1V8PL99c4_7cePrfZ490o9ulvNRMeSnkn?usp=sharing.
